# Blockade of the STAT3/BCL-xL Axis Leads to the Cytotoxic and Cisplatin-Sensitizing Effects of Fucoxanthin, a Marine-Derived Carotenoid, on Human Bladder Urothelial Carcinoma Cells

**DOI:** 10.3390/md23020054

**Published:** 2025-01-22

**Authors:** Wen-Chyi Dai, Tzu-Hsuan Chen, Tzu-Ching Peng, Yung-Ching He, Chao-Yu Hsu, Chia-Che Chang

**Affiliations:** 1 Doctoral Program in Biotechnology Industrial Innovation and Management, National Chung Hsing University, Taichung 402202, Taiwan; d112080201@mail.nchu.edu.tw; 2 Department of Life Sciences, National Chung Hsing University, Taichung 402202, Taiwan; cynthia6616@gmail.com; 3 Graduate Institute of Biomedical Sciences, National Chung Hsing University, Taichung 402202, Taiwan; g113059008@mail.nchu.edu.tw (T.-C.P.); g112059048@mail.nchu.edu.tw (Y.-C.H.); 4 Division of Urology, Department of Surgery, Tungs’ Taichung MetroHarbor Hospital, Taichung 435403, Taiwan; 5 Department of Rehabilitation, Jenteh Junior College of Medicine, Nursing and Management, Miaoli 356006, Taiwan; 6 Master Program in Precision Health, Doctoral Program in Translational Medicine, Rong Hsing Research Center for Translational Medicine, The iEGG and Animal Biotechnology Research Center, National Chung Hsing University, Taichung 402202, Taiwan; 7 Department of Medical Laboratory Science and Biotechnology, Asia University, Taichung 413305, Taiwan; 8 Department of Medical Research, China Medical University Hospital, Taichung 404327, Taiwan

**Keywords:** fucoxanthin, cisplatin, bladder cancer, STAT3, BCL-xL, apoptosis

## Abstract

Bladder cancer is a globally prevalent urological malignancy, with transitional carcinoma (TCC) representing the majority of cases. Cisplatin is the primary drug for metastatic bladder cancer chemotherapy; however, its application is limited by nephrotoxicity and resistance. Signal Transducer and Activator of Transcription 3 (STAT3) is an oncogenic transcription factor often overactivated in various cancers, making it an appealing drug target. Fucoxanthin, a marine carotenoid, has significant anticancer properties. This study explored Fucoxanthin’s cytotoxic effects and its potential to potentiate the efficacy of Cisplatin, along with the mechanisms underlying these effects, on human bladder TCC cells. We demonstrated that Fucoxanthin is cytotoxic to bladder TCC cells by inducing apoptosis, evidenced by z-VAD-fmk-mediated annulment of Fucoxanthin’s cytotoxicity. Furthermore, Fucoxanthin reduced the levels of inherent or interleukin-6-induced tyrosine 705-phosphorylated STAT3 accompanied by downregulating BCL-xL, a well-established STAT3 target. Notably, ectopic expression of STAT3-C, a dominant-active STAT3 mutant, or BCL-xL thwarted Fucoxanthin’s proapoptotic and cytotoxic actions. Moreover, Fucoxanthin at subtoxic dosages enhanced the susceptibility to Cisplatin-induced apoptosis of bladder TCC cells initially resistant to Cisplatin. Remarkably, this Cisplatin-sensitizing effect of Fucoxanthin was abrogated when cells ectopically expressed STAT3-C or BCL-xL. Overall, for the first time, we proved that the proapoptotic, cytotoxic, and Cisplatin-sensitizing effects of Fucoxanthin on human bladder TCC cells are attributed to the blockade of the STAT3/BCL-xL axis. Our findings highlight that targeting the STAT3/BCL-xL axis is a promising strategy to eliminate bladder TCC cells and facilitate Cisplatin sensitization, and further support the potential of incorporating Fucoxanthin into Cisplatin-based chemotherapy for treating bladder cancer.

## 1. Introduction

Bladder cancer is the 10th most commonly diagnosed cancer globally, with men being more likely to develop the disease than women [1]. Urothelial carcinoma, also known as transitional carcinoma (TCC), constitutes over 90% of all bladder cancer cases [2]. Smoking is the most significant modifiable risk factor for bladder cancer, implicated in more than half of all cases [3,4]. Approximately 70–75% of bladder cancer cases are non-muscle-invasive (NMIBC), which has a higher recurrence rate but a lower progression risk. Conversely, muscle-invasive bladder cancer (MIBC) is associated with poorer outcomes and higher metastatic potential [4,5]. Treatment for NMIBC typically involves transurethral resection followed by intravesical Bacillus Calmette-Guérin (BCG) or chemotherapy, while MIBC is treated with radical cystectomy and neoadjuvant chemotherapy [4,5]. Cisplatin-based chemotherapy is recognized as a standard first-line treatment for metastatic bladder cancer [6]. Emerging therapies like immune checkpoint inhibitors and targeted therapeutics offer hope for advanced cases [7,8]. Despite advances in bladder cancer treatment, challenges remain in terms of early detection and reducing recurrence rates [7,8].

Fucoxanthin is a marine-derived carotenoid enriched in brown seaweeds with a broad range of pharmacological activities, including antioxidant, anti-inflammatory, anti-obesity, and anti-cancer effects [9,10]. Its anti-cancer mechanisms involve inducing apoptosis, inhibiting angiogenesis, and modulating key signaling pathways like PI3K/Akt and WNT/β-catenin, highlighting its role in integrative cancer therapies [10,11,12]. Additionally, Fucoxanthin regulates lipid metabolism, reduces fat accumulation, and promotes thermogenesis, making it effective against obesity and metabolic-associated fatty liver disease [13]. Recent studies have also revealed its neuroprotective effects on mitigating oxidative stress and inflammation in neural tissues, underscoring its potential in managing neurodegenerative diseases [14,15].

Signal Transducer and Activator of Transcription 3 (STAT3) is a crucial transcription factor that mediates intracellular signaling in response to cytokines such as interleukin-6 (IL-6) and various growth factors [16]. It plays a key role in regulating a broad range of physiological and pathological processes, including cell proliferation, apoptosis, differentiation, and immune responses [17]. The activation of STAT3 occurs through phosphorylation at the tyrosine 705 residue (Tyr705), which is typically mediated by Janus kinases (JAKs), SRC, or receptor tyrosine kinases (RTKs) after cytokine or growth factor stimulation [18]. Tyr705 phosphorylation allows STAT3 to form dimers and translocate to the nucleus, where it activates the transcription of its target genes, including the gene encoding BCL-xL [18,19]. STAT3 is often overactivated in cancer, contributing to the malignant phenotype by promoting cell survival, angiogenesis, invasion, and immune evasion [20]. Persistent STAT3 signaling has been observed in numerous malignancies, and growing preclinical evidence underscores the effectiveness of targeting STAT3 in the treatment of various cancers, including bladder cancer [21,22,23].

In this study, we investigated the anti-bladder cancer effects of Fucoxanthin and its underlying mechanisms using human bladder TCC cell lines TCCSUP and T24 as our in vitro model. We demonstrated that Fucoxanthin is cytotoxic to bladder TCC cells, and we confirmed that this cytotoxicity depends on the induction of apoptosis. Furthermore, we elucidated that Fucoxanthin induces apoptosis in bladder TCC cells by inhibiting the STAT3/BCL-xL signaling pathway. Notably, we identified that Fucoxanthin enhances the susceptibility of Cisplatin-resistant bladder TCC cells to this chemotherapeutic agent. This sensitizing effect of Fucoxanthin is attributed to the blockade of the STAT3/BCL-xL axis. Overall, our findings support the potential of incorporating Fucoxanthin into Cisplatin-based chemotherapy for the treatment of bladder cancer.

## 2. Results

### 2.1. Fucoxanthin Is More Potent than Cisplatin to Induce Human Bladder TCC Cytotoxicity

**Figure 1:**
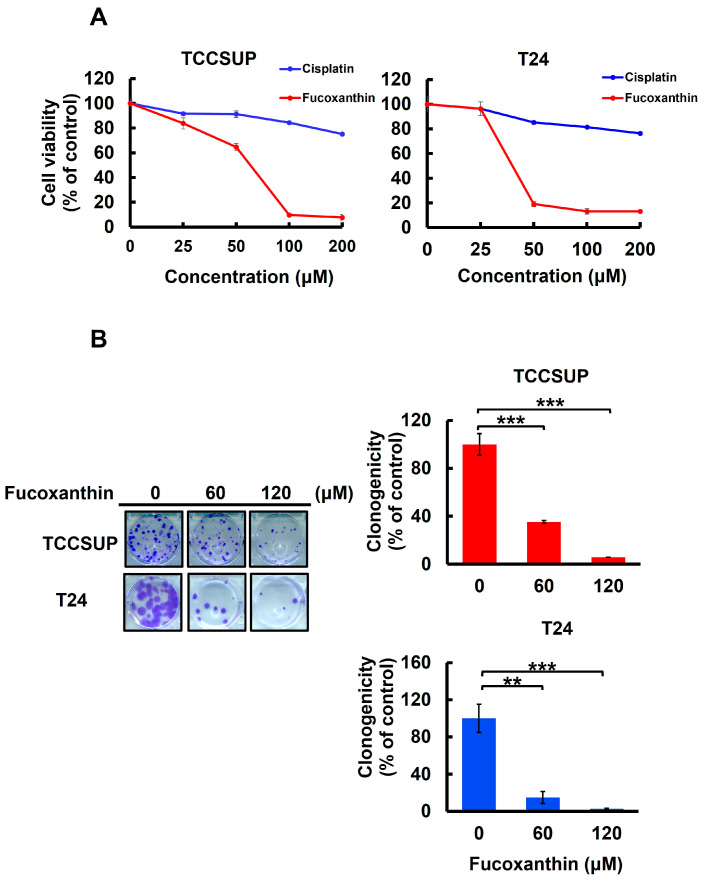
Fucoxanthin’s cytotoxic effect on human bladder TCC cells. (**A**) Cell viability assay. Human bladder TCC cell lines TCCSUP and T24 were treated for 48 h with 0 μM to 200 μM of Fucoxanthin or Cisplatin, a common chemotherapy drug for bladder cancer treatment, followed by cell viability determination using MTT assay as detailed in the Section 4. (**B**) Clonogenicity assay. TCCSUP and T24 cells were treated with Fucoxanthin (0, 60, 120 μM) for 24 h, followed by drug-free incubation for 14 days to form colonies. **: *p* < 0.01; ***: *p* < 0.001.

To assess Fucoxanthin’s anti-bladder cancer potential, we tested the cytotoxic effect of Fucoxanthin on human bladder TCC cell lines TCCSUP and T24 and compared it with Cisplatin, a common chemotherapy agent for bladder cancer treatment. After 48 h treatment with grading doses of each drug (0~200 μM), we found that Fucoxanthin dose-dependently reduced the viability of both cell lines, with an IC_50_ of 63.37 ± 1.04 μM and 39.98 ± 1.33 μM for TCCSUP and T24 cells, respectively. In contrast, Cisplatin showed minimal cytotoxicity, even at the highest dosage of 200 μM (Figure 1A). Additionally, the drop in clonogenicity observed in Fucoxanthin-treated human bladder TCC cells further supported Fucoxanthin’s cytotoxic effects (Figure 1B). Overall, the findings indicated that Fucoxanthin can induce in vitro cytotoxicity against human bladder TCC cells and demonstrated a more significant cytotoxic potential than Cisplatin.

### 2.2. Fucoxanthin-Induced Bladder TCC Cytotoxicity Depends on the Induction of Apoptosis

**Figure 2:**
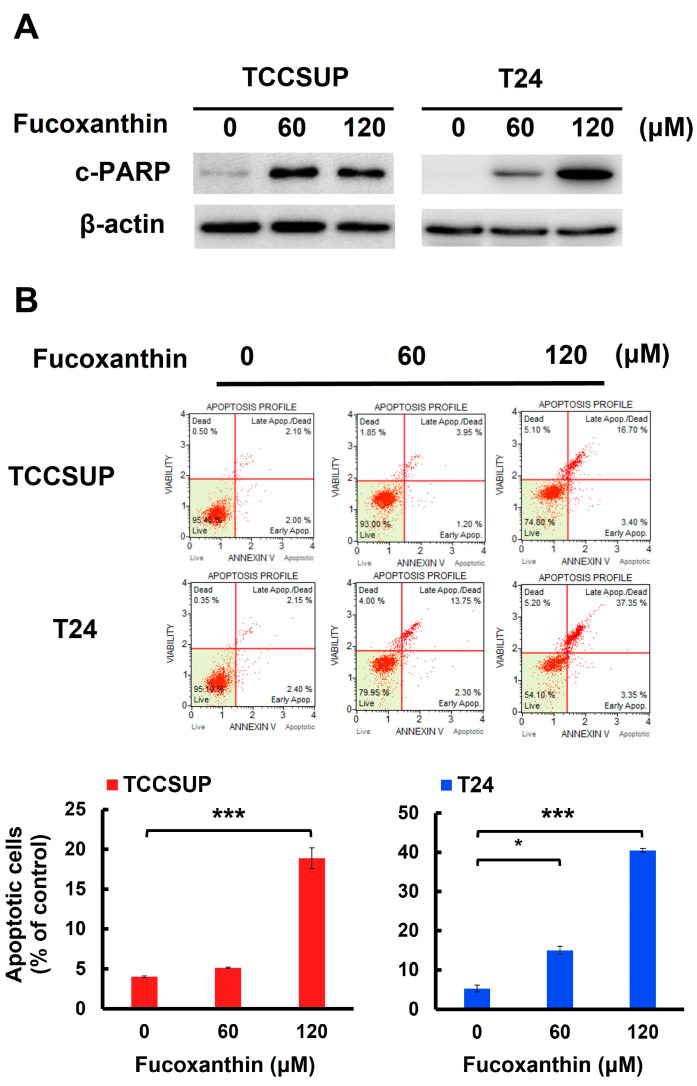

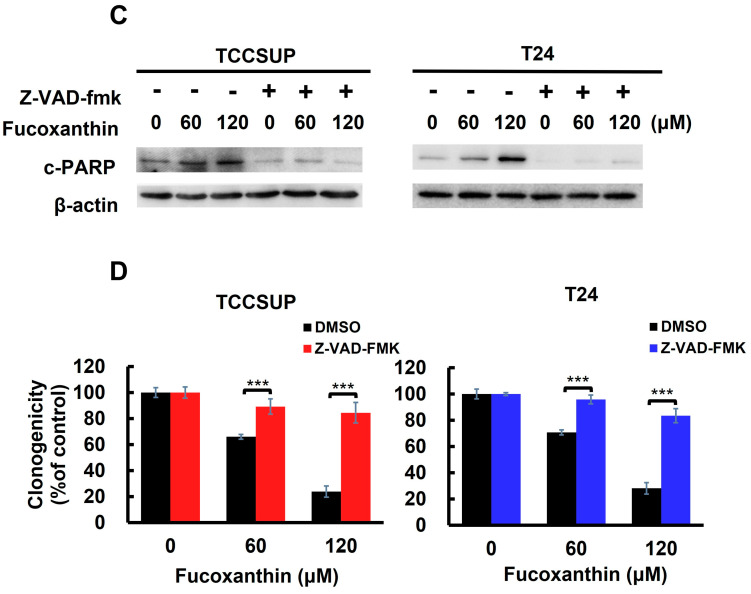
Apoptosis is the primary mechanism of Fucoxanthin’s cytotoxic action on human bladder TCC cells. (**A**) Fucoxanthin dose-dependently triggers PARP cleavage. TCCSUP and T24 cells were treated with Fucoxanthin (0, 60, 120 μM) for 24 h, followed by immunoblotting to detect the levels of cleaved PARP (c-PARP), a canonical biomarker of apoptosis. (**B**) Fucoxanthin increases the levels of annexin V-positively stained cell population. TCCSUP and T24 cells were treated with Fucoxanthin (0, 60, 120 μM) for 24 h, followed by flow cytometry analysis to assess the levels of annexin V-stained (i.e., apoptotic) cell population, represented by scatter plots (upper image) and histograms (lower image). (**C**) Validation of apoptosis blockage by the absence of PARP cleavage. TCCSUP and T24 cells were pre-treated with the pan-caspase inhibitor z-VAD-fmk (50 μM) for 2 h, followed by 24 h treatment with Fucoxanthin. The levels of cleaved PARP were detected using immunoblotting. (**D**) Blockade of apoptosis nullifies Fucoxanthin-induced cytotoxicity. Fucoxanthin-treated TCCSUP and T24 cells with z-VAD-fmk pre-treatment were evaluated for their colony-forming abilities. All immunoblotting used the levels of β-actin as a control for equal loading. *: *p* < 0.05; ***: *p* < 0.001.

We next addressed whether apoptotic cell death is responsible for Fucoxanthin’s cytotoxic effects on human bladder TCC cells. In both TCCSUP and T24 cells, Fucoxanthin treatment led to a significant increase in the levels of cleaved poly (ADP-ribose) polymerase (PARP) (Figure 2A and Figure S1A). This finding suggests that Fucoxanthin induced the activation of caspases, which is a biochemical hallmark of apoptosis induction. Additionally, Fucoxanthin’s proapoptotic effect on human bladder TCC cells was reinforced by a rise in annexin V-positive (apoptotic) cells following Fucoxanthin treatment (Figure 2B). At a concentration of 120 μM, Fucoxanthin significantly increased the percentage of apoptotic cells, from 4.00 ± 0.10% to 18.88 ± 1.28% in TCCSUP cells (*p* < 0.001) and from 5.27 ± 0.88% to 40.52 ± 0.45% in T24 cells (*p* < 0.001), compared to their respective drug-free controls.

With Fucoxanthin’s proapoptotic effect on human bladder TCC cells established, we then examined the functional significance of apoptosis in Fucoxanthin’s cytotoxic action. To investigate this, TCCSUP and T24 cells were pre-treated with a pan-caspase inhibitor z-VAD-fmk to block caspase activation (as shown in Figure 2C and Figure S1B), followed by Fucoxanthin treatment for 24 h and evaluation of clonogenicity afterward. As illustrated in Figure 2D, z-VAD-fmk pre-treatment markedly restored the colony-forming capacity of Fucoxanthin-treated bladder TCC cells. Compared with their respective Fucoxanthin-free controls, z-VAD-fmk pre-treatment restored the clonogenicity of TCCSUP cells from 12.67 ± 4.36% to 81.67 ± 7.90% (*p* < 0.001) and that of T24 cells from 17.33 ± 2.48% to 86.33 ± 5.33% (*p* < 0.001) following 120 μM of Fucoxanthin treatment (Figure 2D). These results supported the induction of apoptosis as a primary mechanism of Fucoxanthin’s cytotoxic action on human bladder TCC cells.

### 2.3. STAT3 Blockade Is Integral to Fucoxanthin’s Cytotoxic Action on Human Bladder TCC Cells

**Figure 3:**
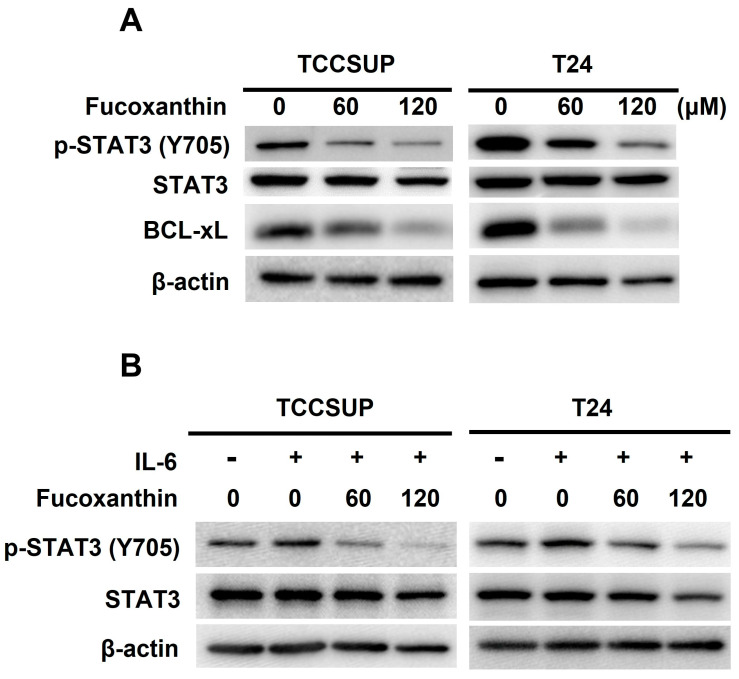

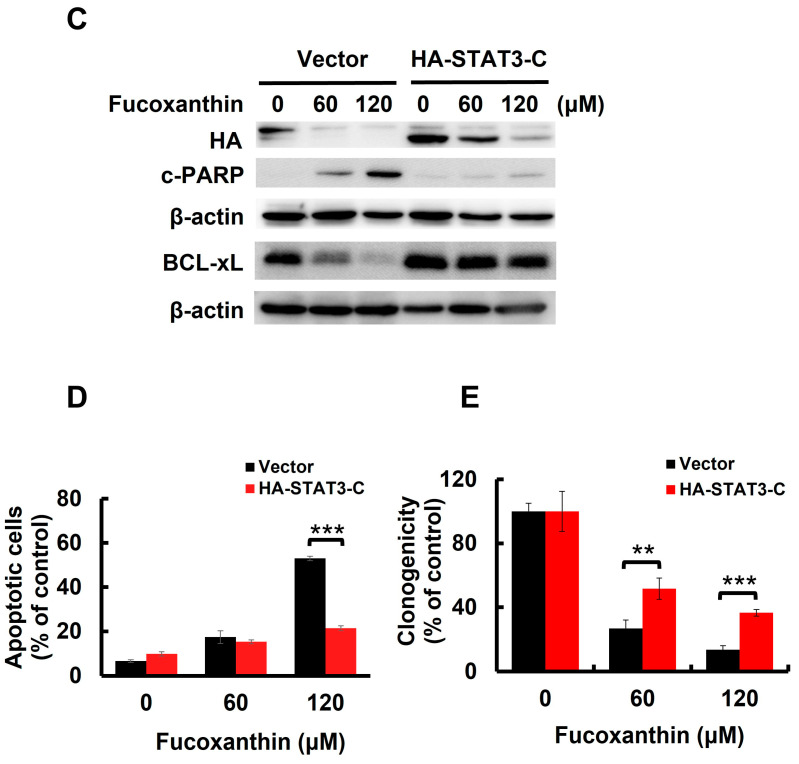
Blockade of STAT3 activation accounts for Fucoxanthin’s proapoptotic and cytotoxic effects on human bladder TCC cells. (**A**) Fucoxanthin inhibits constitutively active STAT3. TCCSUP and T24 cells after 24 h treatment with Fucoxanthin (0, 60, 120 μM) were subjected to immunoblotting for the levels of active STAT3, revealed by Tyr 705 phosphorylation (p-STAT3 (Tyr 705)), and the levels of BCL-xL, a well-known STAT3 transcriptional target. (**B**) Fucoxanthin inhibits IL-6-induced activation of STAT3. TCCSUP and T24 cells were stimulated with IL-6 (100 ng/mL) for 30 min, followed by 24 h treatment with Fucoxanthin and then immunoblotting for p-STAT3 (Tyr 705) levels. (**C**) Ectopic expression of STAT3-C, a dominant-active mutant of STAT3, abolished Fucoxanthin-induced PARP cleavage and BCL-xL downregulation. The vector control and STAT3-C stable clones of T24 cells were treated with Fucoxanthin for 24 h, followed by gauging the levels of c-PARP and BCL-xL using immunoblotting. (**D**) Fucoxanthin failed to enhance annexin V-positive cell population levels of T24 STAT3-C stable clones. The annexin V-stained cells were determined using flow cytometry analysis. (**E**) Fucoxanthin was ineffective in blocking T24 STAT3-C stable clones to form colonies revealed by clonogenicity assay. All immunoblotting used the levels of β-actin as a control for equal loading. **: *p* < 0.01; ***: *p* < 0.001.

The next question we addressed was how Fucoxanthin induces apoptosis in TCCSUP and T24 cells. Given the pro-survival action and oncogenic role of STAT3 in bladder cancer, we explored the role of STAT3 in Fucoxanthin’s proapoptotic effect on these cells. Our immunoblot analysis revealed that Fucoxanthin reduced the levels of tyrosine 705-phosphorylated STAT3 (p-STAT3) along with the downregulation of BCL-xL, a well-recognized transcription target of STAT3 (Figure 3A and Figure S2A). Fucoxanthin’s inhibitory effect on STAT3 was further demonstrated by its ability to prevent the upregulation of p-STAT3 stimulated by IL-6 (Figure 3B and Figure S2B). These lines of evidence suggest that Fucoxanthin has the potential to block STAT3, whether it is constitutively active or induced by IL-6, in human bladder TCC cells.

To further elucidate the significance of STAT3 blockade in Fucoxanthin’s cytotoxicity in bladder TCC, we treated T24 cells stably expressing STAT3-C [24], a dominant-active STAT3 mutant for sustaining STAT3 activation, with Fucoxanthin and analyzed their apoptotic population and clonogenicity. In contrast to promoting PARP cleavage in control clones, Fucoxanthin did not increase cleaved PARP levels in STAT3-C-expressing clones (Figure 3C and Figure S2C). Notably, the reduced levels of PARP cleavage corresponded with a lower apoptotic population (Figure 3D) and increased clonogenicity (Figure 3E) in Fucoxanthin-treated STAT3-C-expressing cells. Overall, these results identify the blockade of STAT3 activation as a pivotal mechanism underlying the proapoptotic action of Fucoxanthin, ultimately contributing to its cytotoxic effects on bladder TCC.

### 2.4. BCL-xL Downregulation Caused by STAT3 Blockade Mediates Fucoxanthin-Induced Human Bladder TCC Cytotoxicity

**Figure 4:**
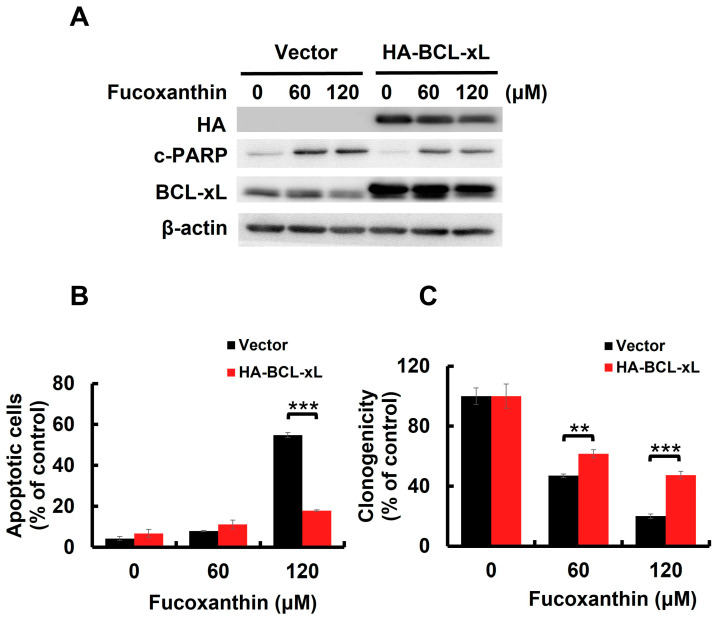
BCL-xL downregulation accounts for Fucoxanthin’s proapoptotic and cytotoxic effects on human bladder TCC cells. (**A**) Ectopic BCL-xL expression sabotages Fucoxanthin-induced PARP cleavage. The vector control and BCL-xL stable clones of T24 cells were treated with Fucoxanthin for 24 h, then were gauged for the levels of c-PARP and BCL-xL using immunoblotting. (**B**) T24 BCL-xL stable clones resist Fucoxanthin-induced upregulation of annexin V-positive cell population, assessed by flow cytometry analysis. (**C**) T24 BCL-xL stable clones exhibit resilience against Fucoxanthin’s inhibitory effect on colony-forming capacity. All immunoblotting used the levels of β-actin as a control for equal loading. **: *p* < 0.01; ***: *p* < 0.001.

BCL-xL is a potent antiapoptotic BCL-2 family protein [25]. We previously found that Fucoxanthin downregulated BCL-xL in both TCCSUP and T24 cells (Figure 3A and Figure S2A). However, STAT3-C overexpression annulled Fucoxanthin-induced BCL-xL downregulation (Figure 3C), confirming STAT3 as a positive upstream regulator of BCL-xL expression. We then investigated whether the downregulation of BCL-xL, following STAT3 inhibition by Fucoxanthin, contributes to the proapoptotic and cytotoxic effects of Fucoxanthin on human bladder TCC cells. To explore this issue, we treated T24 clones that stably express BCL-xL (refer to Figure 4A and Figure S3A,B) with Fucoxanthin, subsequently evaluating their apoptotic status and clonogenicity. Notably, BCL-xL overexpression allowed these cells to resist Fucoxanthin-induced apoptosis (Figure 4B and Figure S3C) and to maintain their clonogenicity (Figure 4C) compared to the control clones. These findings support the hypothesis that Fucoxanthin disrupts STAT3 activation to downregulate BCL-xL, ultimately leading to the induction of apoptosis and resulting cytotoxicity in human bladder TCC cells.

### 2.5. Fucoxanthin Blocks the STAT3/BCL-xL Signaling Axis to Facilitate Cisplatin Sensitization of Human Bladder TCC Cells

**Figure 5:**
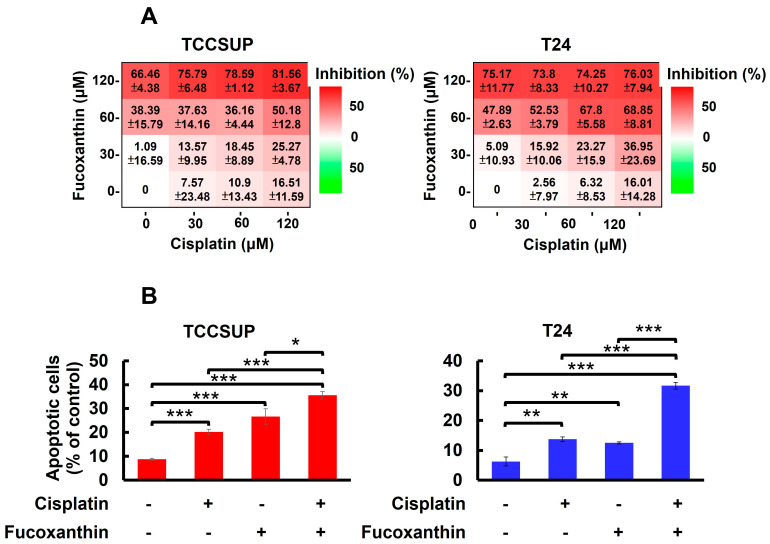

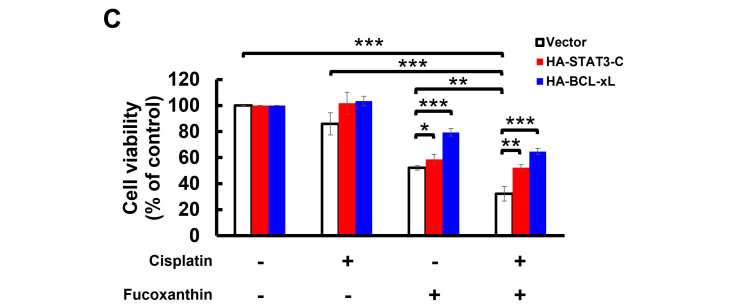
Fucoxanthin potentiates Cisplatin’s efficacy in human bladder TCC cells initially resistant to Cisplatin by blocking the STAT3/BCL-xL axis. (**A**) TCCSUP and T24 cells were treated for 24 h with Fucoxanthin (0, 30, 60, 120 μM), Cisplatin (0, 30, 60, 120 μM), or combinations of Fucoxanthin with Cisplatin, followed by gauging the percentage of viability inhibition compared to drug-free controls using MTT assay. (**B**) Fucoxanthin at a subtoxic dosage significantly enhances the levels of Cisplatin-induced apoptosis. TCCSUP cells were treated for 24 h with Fucoxanthin (60 μM), Cisplatin (120 μM), or a combination of Fucoxanthin with Cisplatin, then the levels of annexin V-positive (apoptotic) cell populations were scored by flow cytometry analysis. Likewise, combined treatment with Fucoxanthin (60 μM) markedly uplifts the apoptotic cell population of T24 cells triggered by Cisplatin (60 μM) treatment alone. (**C**) Ectopic expression of STAT3-C or BCL-xL negates the Cisplatin-sensitizing effect of Fucoxanthin. The vector control, STAT3-C, or BCL-xL stable clones of T24 cells were subjected to 24 h treatment with Fucoxanthin (60 μM), Cisplatin (60 μM), or the combination of both. MTT assay was then employed to assess the percentage of cell survival relative to the viability of the vector control under these treatments. *: *p* < 0.05; **: *p* < 0.01; ***: *p* < 0.001.

In Figure 1A, we showed that TCCSUP and T24 cells were refractory to Cisplatin treatment, with over 80% of cells still alive even with the drug dosages reaching 200 μM. Accordingly, we aimed to test whether co-treatment with Fucoxanthin sensitizes bladder TCC cells to the cytotoxic effects of Cisplatin. Our findings revealed that treating TCCSUP cells with either Fucoxanthin (60 μM) or Cisplatin (120 μM) alone for 24 h inhibited cell viability by 38.39 ± 15.79% or 16.51 ± 11.59% of the drug-free control, respectively. However, when the two treatments were combined, Fucoxanthin markedly potentiated Cisplatin’s efficacy, increasing cell viability inhibition to 50.18 ± 12.80% (Figure 5A, left). For T24 cells, the effect of Cisplatin (60 μM) on cell viability was drastically enhanced from 6.32 ± 8.53% when applied alone to 67.80 ± 5.58% when co-treated with Fucoxanthin (60 μM) (Figure 5A, right). Notably, the increased levels of cell viability inhibition observed with the combination of Fucoxanthin and Cisplatin coincided with elevated rates of apoptotic cell death (Figure 5B). Together, these findings underscore the significant potential of Fucoxanthin to enhance the susceptibility of human bladder TCC cells to Cisplatin-induced cytotoxicity, primarily through the induction of apoptosis.

Considering Fucoxanthin’s inhibitory effect on STAT3 activation (refer to Figure 3) and the potent antiapoptotic action of STAT3 signaling, we aimed to decipher the role of STAT3 signaling in Fucoxanthin-facilitated Cisplatin sensitization. To this end, T24 cells stably expressing STAT3-C were treated for 24 h with Cisplatin (60 μM), Fucoxanthin (60 μM), or a combination of Cisplatin (60 μM) with Fucoxanthin (60 μM), followed by determining cell viability under these treatment conditions. We found that cell viability was significantly decreased following combination treatments, in contrast to treatments with either drug alone (*p* < 0.001) (Figure 5C, white bars). Notably, the enhancement of Cisplatin cytotoxicity by Fucoxanthin was abrogated in the STAT3-C stable clones (*p* < 0.01) (Figure 5C, red bars). Likewise, Fucoxanthin failed to sensitize Cisplatin in T24 cells overexpressing BCL-xL (*p* < 0.001) (Figure 5C, blue bars). Together, these findings argued that inhibiting the STAT3/BCL-xL axis accounts for the mechanism whereby Fucoxanthin facilitates Cisplatin sensitization.

## 3. Discussion

We herein uncovered that blocking the STAT3/BCL-xL axis serves as the mechanism whereby Fucoxanthin exerts its proapoptotic and cytotoxic effects on human bladder TCC cells as well as its ability to sensitize these cells to Cisplatin. Specifically, we showed that Fucoxanthin is more effective than Cisplatin in exerting bladder TCC cytotoxicity (Figure 1), mainly by promoting apoptosis-dependent cell death (Figure 2). Next, we unraveled Fucoxanthin’s inhibitory effect on STAT3 activation, which led to BCL-xL downregulation (Figure 3). We further proved that blocking STAT3 or downregulating BCL-xL is central to Fucoxanthin’s proapoptotic and cytotoxic actions (Figure 3 and Figure 4). Notably, we revealed that Fucoxanthin sensitizes Cisplatin in bladder TCC cells initially resistant to this chemotherapy drug and further elucidated that this Cisplatin-sensitizing effect of Fucoxanthin occurs through the inhibition of the STAT3/BCL-xL axis (Figure 5). To our knowledge, these findings have never been reported previously.

In this study, we argued that Fucoxanthin provokes bladder TCC cell death primarily through the activation of apoptosis, as evidenced by the nearly complete restoration of clonogenicity observed when apoptosis was inhibited by z-VAD-fmk (Figure 2). Previous studies by Zhang et al. [26] and Wang et al. [27] also reported Fucoxanthin’s proapoptotic effect on human bladder TCC cell lines EJ-1 and T24, respectively. However, those studies did not clarify whether the induction of apoptosis is critical for Fucoxanthin’s cytotoxic effect on bladder TCC cells, as we have explored in this study. Besides triggering apoptosis, Fucoxanthin was found to cause cell cycle arrest at the G_0_/G_1_ phase in T24 cells by upregulating p21^CIP1^, ultimately reducing clonogenicity [27]. In view of that, our future studies will examine Fucoxanthin’s effects on cell cycle progression in additional bladder TCC cell lines.

The current study highlights the central role of STAT3 inhibition in Fucoxanthin’s proapoptotic and cytotoxic actions on bladder TCC cells. Numerous pieces of evidence have identified the aberrant activation of STAT3 as a critical driver of various aspects of malignant progression in bladder cancer, including cell proliferation [28], epithelial-to-mesenchymal transition [29,30], angiogenesis [31], cancer metabolic reprogramming [32,33], cancer stemness maintenance [34,35], and evasion of immunosurveillance [36,37]. Furthermore, multiple in vitro and preclinical studies have demonstrated the therapeutic benefits of pharmacologically blocking STAT3 to treat bladder cancer [22,23]. Thus, our current findings underpin STAT3 blockage as a potential strategy for bladder cancer treatment. It is also noteworthy that Wang et al. proclaimed that Fucoxanthin induces T24 cell apoptosis by downregulating mortalin, a member of the Hsp70 protein family with oncogenic function [27,38]. Interestingly, Teng et al. demonstrated that one of mortalin’s oncogenic roles involves activating STAT3, which enhances the motility and invasiveness of human hepatocellular carcinoma cells [39]. In light of this, it would be intriguing to investigate whether Fucoxanthin targets mortalin to inhibit STAT3 activation.

For the first time, we demonstrated that Fucoxanthin at subtoxic dosages potentiates Cisplatin’s toxicity in bladder TCC cells initially resistant to Cisplatin, likely by promoting Cisplatin-elicited apoptotic responses (Figure 5A,B). Cisplatin is the primary chemotherapeutic agent used for treating locally advanced and metastatic bladder cancer [40]. However, its use is limited due to several toxic side effects, including nephrotoxicity, neurotoxicity, and cardiotoxicity [41]. Additionally, intrinsic and acquired resistance developed in cancer cells often sabotages Cisplatin’s effectiveness, leading to therapeutic failure [42]. Numerous in vitro and in vivo studies have highlighted the potential benefits of combining Cisplatin with other drugs to achieve greater efficacy, reduced toxicity, and decreased chemoresistance compared to Cisplatin alone [42,43]. Our findings position Fucoxanthin as a promising candidate for Cisplatin-based combination therapies [42,43].

The data presented here indicate that blocking the STAT3/BCL-xL axis contributes to the Cisplatin-sensitizing effects of Fucoxanthin (Figure 5C). The activation of STAT3 has been identified as a key mechanism for conferring survival advantage on cancer cells in response to the toxic effects of chemotherapeutics, such as Cisplatin and tyrosine kinase inhibitors [40,44]. Additionally, growing evidence has underscored that targeting STAT3 could effectively overcome Cisplatin resistance in various types of cancer cells [45,46,47,48,49,50]. Furthermore, as a well-recognized transcriptional target of STAT3, BCL-xL expression has been identified as a poor prognostic marker for bladder cancer [51]. Notably, it has been proven that the survival advantage conferred by BCL-xL expression is pivotal to sustaining Cisplatin resistance in ovarian, lung, and oral cancer cells while downregulating BCL-xL sensitizes these cells effectively to the toxic effects of Cisplatin [52,53,54]. Thus, our findings reinforce the potential of targeting the STAT3/BCL-xL axis as a promising strategy to reverse Cisplatin resistance.

One of the questions that remains to be addressed is how Fucoxanthin inhibits the activation of STAT3. Our previous study on melanoma cells showed that Fucoxanthin targets JAK2, an upstream kinase responsible for inducing the phosphorylation of STAT3 at tyrosine 705 [55]. In the context of bladder TCC cells, we found that Fucoxanthin lowered the levels of tyrosine 1007/1008-phosphorylated JAK2 (p-JAK2) as well as total JAK2 in both TCCSUP and T24 cells (Figure S4A). Regarding SRC, another upstream kinase of STAT3, Fucoxanthin downregulated tyrosine 416-phosphorylated SRC (p-SRC) along with total SRC in both cell lines (Figure S4B). Interestingly, while Fucoxanthin increased the ratios of p-JAK2 to total JAK2 and p-SRC to total SRC in TCCSUP cells, these ratios decreased in T24 cells (Figure S4A,B). The inconsistent effects of Fucoxanthin on JAK2 and SRC suggest the likely involvement of additional mechanisms in Fucoxanthin-elicited blockade of STAT3 activation. Besides positive regulators like JAK2 and SRC, the activation of STAT3 is negatively regulated by protein tyrosine phosphatases like Src homology region 2 domain-containing phosphatase (SHP)-1 and SHP-2 [56,57], protein inhibitors of activated STAT3 (PIAS3) [58], and the suppressor of cytokine signaling-3 (SOCS3) [59]. Our follow-up investigation will explore whether Fucoxanthin engages these negative regulators of STAT3 to block its activation. Alternatively, the possibility that Fucoxanthin directly binds to STAT3 and inhibits its activation cannot be excluded. We will conduct molecular docking analysis to address this issue in the future.

The findings shown here revealed Fucoxanthin’s cytotoxic and Cisplatin-sensitizing effects and supported the central role of STAT3/BCL-xL axis blockage underlying these effects. However, it should be noted that these conclusions were based entirely on in vitro studies using human bladder TCC cell lines. The potential of Fucoxanthin in bladder cancer treatment could be further underpinned by solid lines of in vivo evidence, including Fucoxanthin-induced suppression of xenografted bladder tumor growth, decreased levels of p-STAT3 and BCL-xL in Fucoxanthin-treated tumor tissues, and enhanced growth retardation of Cisplatin-treated bladder tumors when combined with Fucoxanthin. Accordingly, the in vivo establishment of Fucoxanthin’s anti-bladder potential will be a key focus of our future studies.

**Figure 6:**
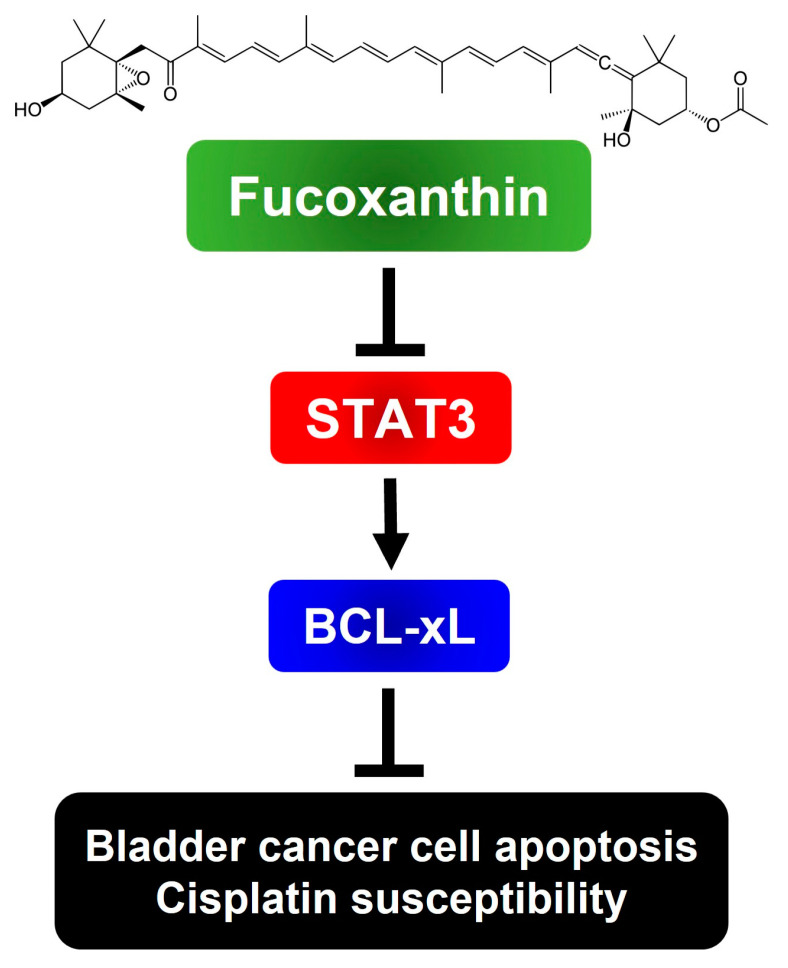
Schematic model of the cytotoxic and Cisplatin-sensitizing effects of Fucoxanthin on human bladder TCC cells elucidated in this study. To exert its cytotoxic effect on human bladder TCC cells, Fucoxanthin suppresses the antiapoptotic STAT3/BCL-xL signaling axis to trigger apoptosis, leading to the elimination of cancer cells and sensitization of Cisplatin efficacy. The image of Fucoxanthin was acquired from Wikipedia. (Image source: Fucoxanthin (2 September 2024) in Wikipedia, https://en.wikipedia.org/wiki/Fucoxanthin)

To sum up, we have, for the first time, revealed the blockade of the STAT3/BCL-xL axis as the mechanism by which Fucoxanthin exerts proapoptotic and cytotoxic effects, while also enhancing Cisplatin sensitization in human bladder TCC cells (Figure 6). Our findings suggest the potential of integrating Fucoxanthin into Cisplatin-based combination therapy regimens to treat bladder cancer.

## 4. Materials and Methods

### 4.1. Chemicals and Reagents

Fucoxanthin was purchased from Cayman Chemical (Ann Arbor, MI, USA; Cat. No. 13068). Cisplatin was acquired from AdooQ^®^ Bioscience (Irvine, CA, USA). Both Fucoxanthin and Cisplatin were prepared as a 20 mM stock solution in dimethyl sulfoxide (DMSO) and stored at −20 °C before use. Recombinant human interleukin-6 (IL-6) was obtained from PeproTech (Rehovot, ISR). Methylthiazolyldiphenyl-tetrazolium bromide (MTT) was bought from Sigma-Aldrich (St. Louis, MO, USA). All reagents for cell culture, including Minimum Essential Medium (MEM), McCoy’s 5A medium, fetal bovine serum (FBS), sodium pyruvate, and penicillin–streptomycin (P/S), were purchased from Gibco Life Technologies (Carlsbad, CA, USA).

### 4.2. Cell Culture

Human urinary bladder TCC cell lines TCCSUP (ATCC HTB-5™) and T24 (ATCC HTB-4™) were purchased from the American Type Culture Collection (ATCC) (Manassas, VA, USA) and Bioresource Collection and Research Center (Hsinchu, Taiwan), respectively. TCCSUP was cultured in MEM with 1 mM sodium pyruvate. T24 cells were cultured in McCoy’s 5A medium. Both culture media were supplemented with non-essential amino acids, 1 mM sodium pyruvate, 10% fetal bovine serum, and 1% penicillin–streptomycin. All cell cultures were incubated at 37 °C in a humidified atmosphere with 5% CO_2_.

### 4.3. Cytotoxicity Assay

Cell viability was assessed using the MTT assay. In brief, TCCSUP and T24 cells were seeded at a density of 8 × 10^3^ cells per well in 96-well plates and treated with Fucoxanthin at concentrations ranging from 0 μM to 200 μM for 24 h and 48 h. Following treatment, 10 μL of MTT reagent (5 mg/mL) was added to each well, and cells were incubated for 4 h. Formazan crystals were dissolved in isopropanol, and absorbance was measured at 563 nm using a Tecan Sunrise™ absorbance reader. Viability was expressed as a percentage of 563 nm absorbance of drug-treated groups relative to drug-untreated controls.

### 4.4. Clonogenicity Assay

To evaluate the long-term cytotoxic effect of Fucoxanthin, TCCSUP and T24 cells after 24 h treatment with Fucoxanthin (0, 60, 120 μM) were seeded in 6-well plates at a density of 5 × 10^2^ cells per well. Then, cells were grown in drug-free media for 12–14 days to form colonies. Colonies were fixed with methanol, stained with 2% crystal violet, and manually scored by at least two individuals.

### 4.5. Apoptosis Assay

We utilized the Muse^®^ Annexin V & Dead Cell Assay Kit (Millipore; Burlington, MA, USA) to assess the proapoptotic effects of Fucoxanthin and Cisplatin on TCCSUP and T24 cells following our established procedures [60]. In short, cells (3 × 10^5^ cells per well on a 6-well plate) were treated with Fucoxanthin (0, 60, 120 μM) for 24 h. After treatment, the cells were resuspended by trypsinization and washed twice with phosphate-buffered saline (PBS). Subsequently, they were incubated for 20 min at room temperature in the dark with 100 μL of Annexin V & Dead Cell reagent. The levels of annexin V-positive (apoptotic) cell populations were then determined by flow cytometry on the Muse^®^ Cell Analyzer (Millipore; Burlington, MA, USA).

### 4.6. Plasmids and Stable Clone Establishment

The plasmids pBabe-HA-STAT3-C and pBabe-HA-BCL-xL are based on the pBabe mammalian expression vector system, designed for the ectopic expression of the N-terminal hemagglutinin (HA) epitope-tagged STAT3-C, a dominant-active mutant of STAT3 (specifically, STAT3 (A662C/N664C)), and human BCL-xL. The construction strategy for these plasmids has been thoroughly detailed in our previous report [60]. We established clones that stably express HA-STAT3-C or HA-BCL-xL proteins in the T24 cell line using our established protocol [60]. Ectopic expression of these proteins was confirmed by immunoblotting.

### 4.7. Immunoblotting

Proteins were extracted from cells using RIPA buffer containing protease and phosphatase inhibitors (Roche, Basel, CHE). About 20~30 μg of protein samples were resolved by SDS-PAGE and transferred onto PVDF membranes (Millipore; Burlington, MA, USA). The primary antibodies against HA epitope (#3724), JAK2 (#3230), cleaved PARP (#9451), SRC (#2108), p-SRC (Tyr 416) (#6943), STAT3 (#12640), and p-STAT3 (Tyr 705) (#9131) were all obtained from Cell Signaling Technology (Boston, MA, USA). The BCL-xL antibody (10783-1-AP) was purchased from Proteintech (Rosemont, IL, USA). Additional antibodies detecting p-JAK2 (Tyr1007/1008) (GTX132784) and β-actin (GTX109639) were bought from GeneTex (Irvine, CA, USA). Secondary antibodies were procured from Jackson ImmunoResearch Laboratories (West Grove, PA, USA). Blot signals were detected by enhanced chemiluminescence (ECL) reagents (Millipore; Burlington, MA, USA).

### 4.8. Statistical Analysis

All experiments were performed in triplicates. Data were presented as mean ± standard deviation (SD). Statistical significance was evaluated using Student’s *t*-test. A *p*-value lower than 0.05 was considered statistically significant.

## Data Availability

All data produced or analyzed during this study are presented in this published article.

## Supplementary Materials

The following supporting information can be downloaded at: www.mdpi.com/article/10.3390/md23020054/s1, Figure S1: Quantitative results of the density of the proteins shown in the immunoblot images in Figure 2. Figure S2: Quantitative results of the density of the proteins shown in the immunoblot images in Figure 3. Figure S3: Quantitative results of the density of the proteins shown in the immunoblot images in Figure 4C. Figure S4: Inconsistent effects of Fucoxanthin on the activation of JAK2 or SRC in TCCSUP and T24 cells.
