# Supplementary material for: Blockade of the STAT3/BCL-xL Axis Leads to the Cytotoxic and Cisplatin-Sensitizing Effects of Fucoxanthin, a Marine-Derived Carotenoid, on Human Bladder Urothelial Carcinoma Cells
Source: Mar Drugs. 2025 Jan 22;23(2):54. doi: 10.3390/md23020054 (PMC11857094; doi:10.3390/md23020054)
Supplement: Supplementary file 1 [file marinedrugs-23-00054-s001.zip › marinedrugs-3428524-supplementary.pdf]

marine drugs

MDPI

Article

Blockade of the STAT3/BCL-xL Axis Leads to the Cytotoxic and Cisplatin-Sensitizing Effects of Fucoxanthin, A Marine-Derived Carotenoid, on Human Bladder Urothelial Carcinoma Cells

Wen-Chyi Dai [1], Tzu-Hsuan Chen [2,†], Tzu-Ching Peng [3,†], Wen-Liang Chang [4], Yung-Ching He [3], Chao-Yu Hsu [5,6,*], and Chia-Che Chang [1,2,3,7,8,9,*]

Supplementary Figures

Figure S1

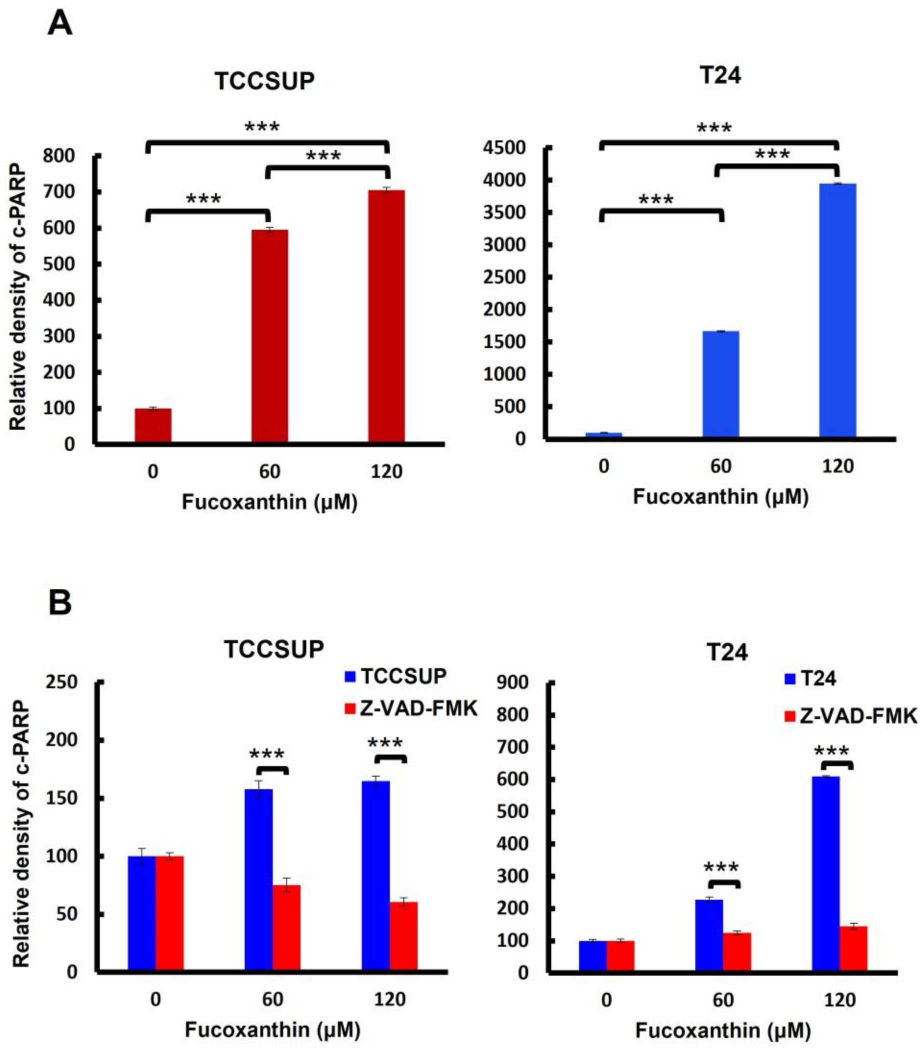

Figure S1. Quantitative results of the density of the proteins shown in the immunoblot images in Figure 2. (**A**) Dose-dependent elevation in the levels of cleaved PARP (c-PARP) in Fucoxanthin-treated human bladder transitional cell carcinoma (TCC) cell lines TCCSUP and T24 shown in **Figure 2A**. (**B**) Blockade of Fucoxanthin-mediated increase in c-PARP levels when TCCSUP and T24 cells were pretreated with the pan-caspase inhibitor z-VAD-fmk shown **Figure 2C**. All immunoblot analyses used β-actin levels as the control for equal loading. The extents of the c-PARP-to-β-actin ratio of Fucoxanthin-treated cells relative to drug-free controls on the blots were quantified using the ImageJ algorithm. ***: $p < 0.001$.

Figure S2

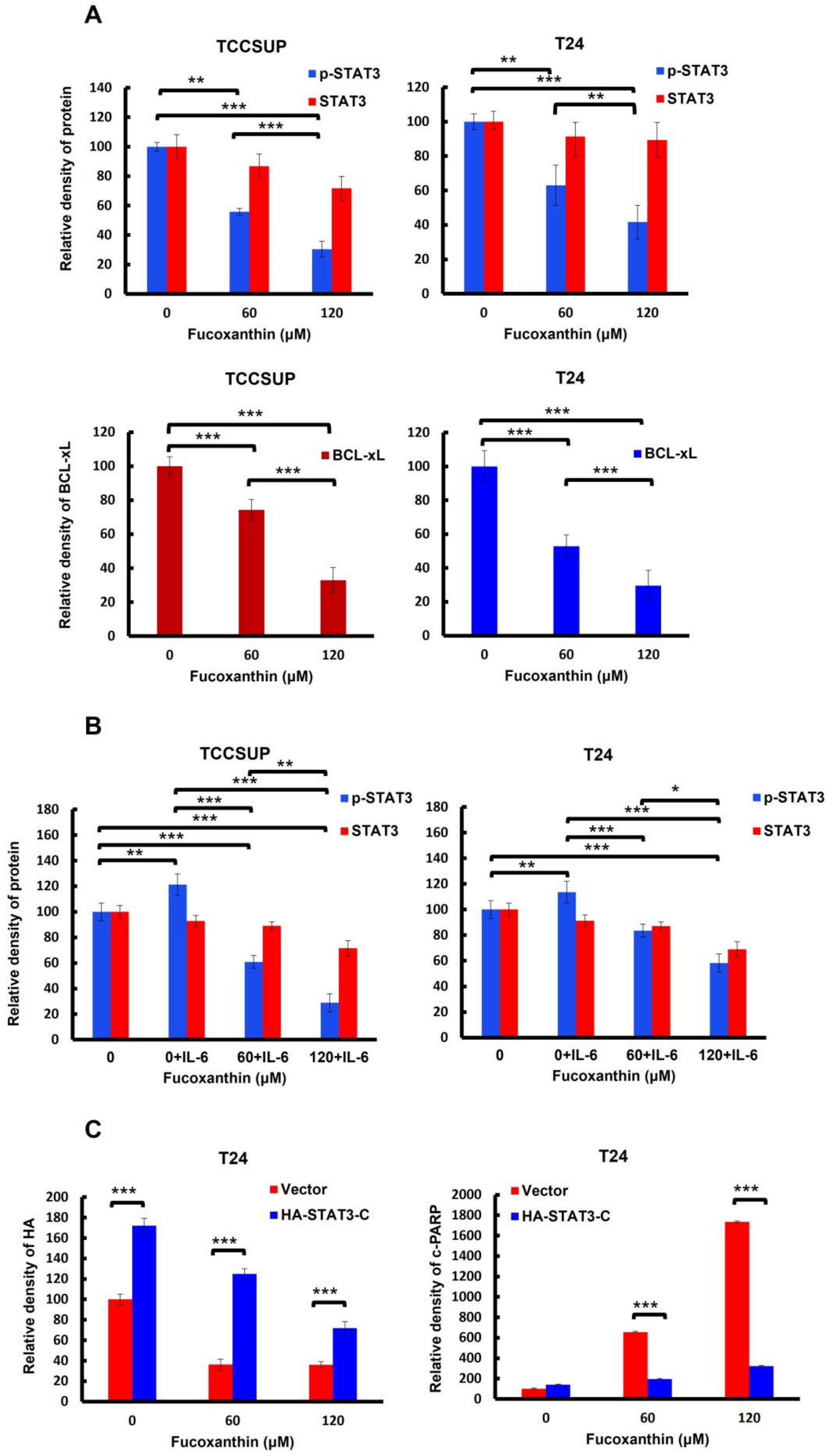
A
TCCSUP
T24
p-STAT3
STAT3
Relative density of protein
Fucoxanthin (μM)
Relative density of BCL-xL
BCL-xL
B
0+IL-6
60+IL-6
120+IL-6
C
Vector
HA-STAT3-C
Relative density of HA
Relative density of c-PARP

Figure S2. Quantitative results of the density of the proteins shown in the immunoblot images in Figure 3.
(**A**) Dose-dependent decrease in the levels of tyrosine 705-phosphorylated STAT3 (p-STAT3) along with BCl-xL, a well-known STAT3 transcriptional target, in Fucoxanthin-treated TCCSUP and T24 cells shown in **Figure 3A**. (B) Fucoxanthin inhibits IL-6-induced up-regulation of p-STAT3 in TCCSUP and T24 cells shown in **Figure 3B**. (**C**) Blockade of Fucoxanthin-mediated increase in c-PARP levels in TCCSUP and T24 cells with stable expression of STAT3-C, a dominant-active STAT3 mutant shown in **Figure 3C**. All immunoblot analyses used β-actin levels as the control for equal loading. The extents of the proteins-to-β-actin ratio of Fucoxanthin-treated cells relative to drug-free controls on the blots were quantified using the ImageJ algorithm. **: $p < 0.01$; ***: $p < 0.001$.

Figure S3

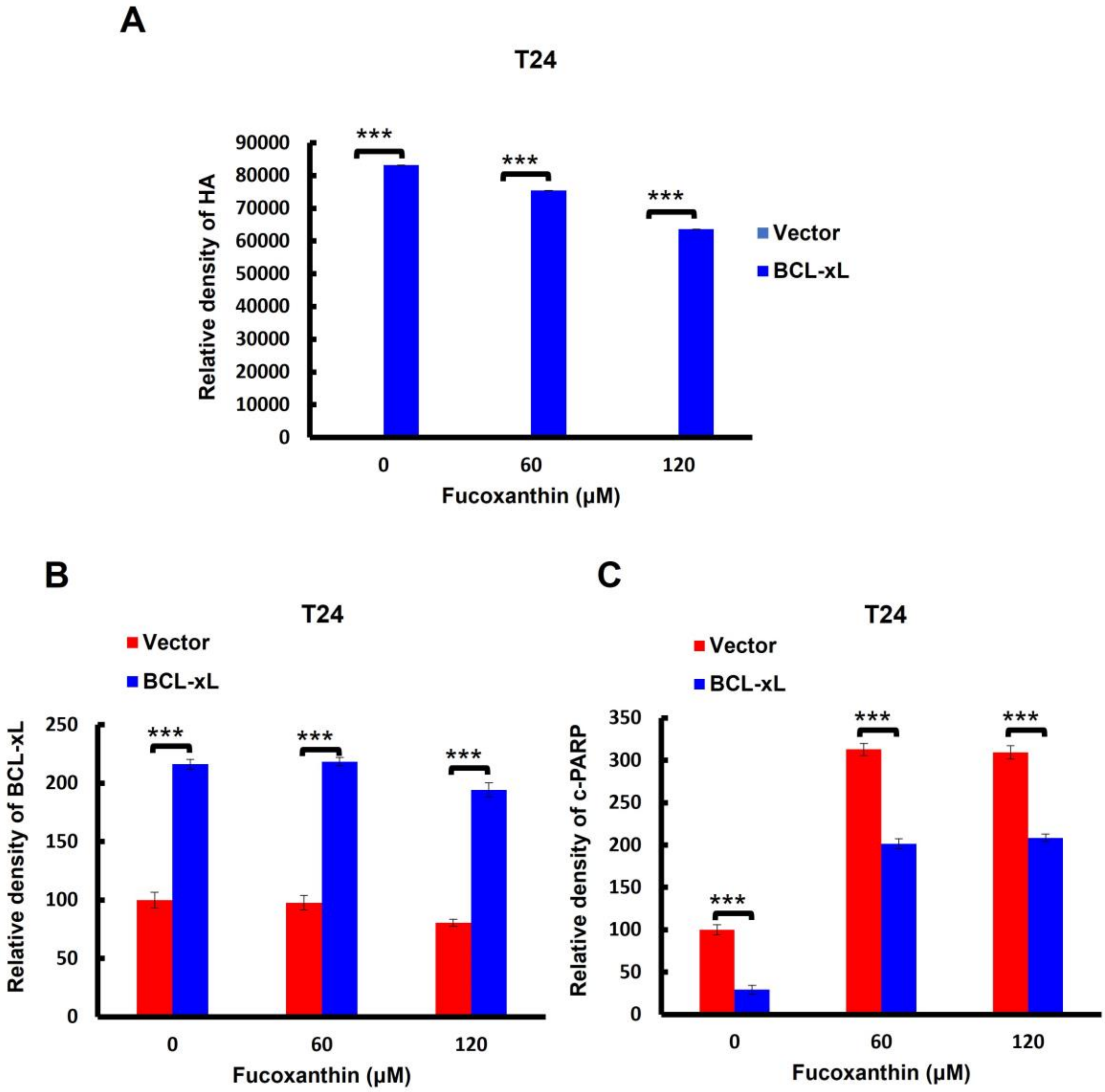

Figure S3. Quantitative results of the protein density shown in the immunoblot images in Figure 4C Overexpression of HA-BCL-xL in T24 cells was revealed by the increased levels of hemagglutinin (HA) (**A**) or BCL-xL (**B**). Additionally, BCL-xL overexpression markedly attenuated Fucoxanthin-mediated upregulation of c-PARP (**C**). All immunoblot analyses used β-actin levels as the control for equal loading. The extents of the proteins-to-β-actin ratio of Fucoxanthin-treated cells relative to drug-free controls on the blots were quantified using the ImageJ algorithm. ***: $p < 0.001$.

Figure S4

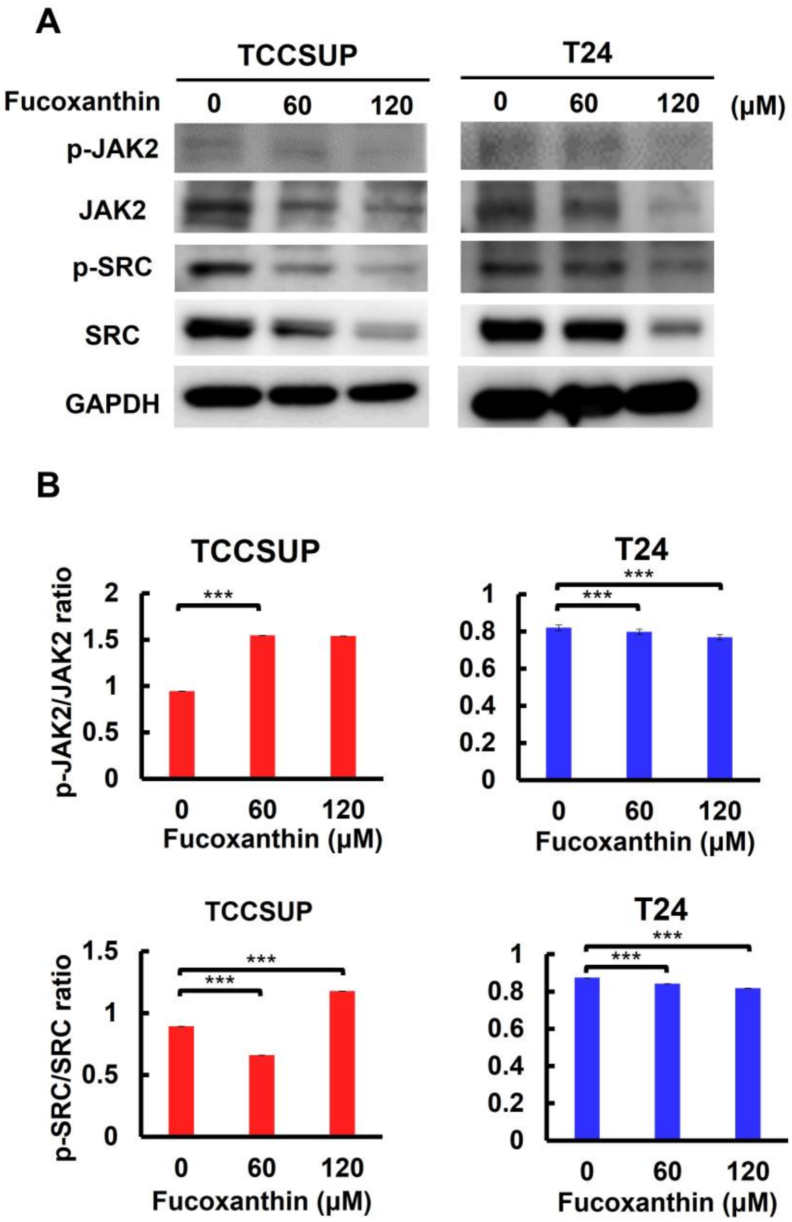

Figure S4. Inconsistent effects of Fucoxanthin on the activation of JAK2 or SRC in TCCSUP and T24 cells. To examine whether Fucoxanthin targets JAK2 and/or SRC to interfere with STAT3 activation in human bladder transitional cell carcinoma (TCC) cells, TCCSUP and T24 cells were treated for 24 h with Fucoxanthin (0, 60, 120 μM), followed by immunoblotting for the levels of active JAK2 (p-JAK2; phosphor-JAK2 (Tyr 1007/1008)), total JAK2, active SRC (p-SRC; phosphor-SRC (Tyr 416)), and total SRC. The levels of GAPDH were used as the control for equal loading. The results showed that Fucoxanthin reduced the levels of both active JAK2 and SRC, as well as their respective total protein levels (**Figure S1A**). However, the ratios of p-JAK2/JAK2 and p-SRC/SRC exhibited contrasting trends: Fucoxanthin enhanced these ratios in TCCSUP cells while decreasing them in T24 cells (**Figure S1B**). These findings suggest that Fucoxanthin's effects on JAK2 and SRC activation are context-dependent and vary between different cell types. ***: $p < 0.001$.
